# Restoration of vision by combined experimental antithymocyte therapy, and orbital radiation with high-dose steroids for severe, acute, steroid-refractory, congestive thyroid orbitopathy

**DOI:** 10.1007/s10633-023-09955-6

**Published:** 2023-09-29

**Authors:** Monika Sarnat-Kucharczyk, Dorota Pojda-Wilczek, Maria Świerkot, Grażyna Kulawik, Ewa Mrukwa-Kominek

**Affiliations:** 1 Department of Ophthalmology, Faculty of Medical Sciences in Katowice, Medical University of Silesia, Katowice, Poland; 2 Kornel Gibinski University Clinical Centre, Katowice, Poland; 3 Department of Internal Medicine and Oncological Chemotherapy, Faculty of Medical Sciences in Katowice, Medical University of Silesia, Katowice, Poland; 4 Andrzej Mielecki Clinical Hospital, Katowice, Poland

**Keywords:** Optic neuropathy, Graves’ Orbitopathy, Exophthalmos, Electrophysiological examinations

## Abstract

**Purpose:**

We report diagnostic and therapeutic dilemmas in the difficult case of compressive optic neuropathy with severe visual acuity and visual field loss with subsequent visual recovery in both eyes, in a patient with Graves’ orbitopathy (GO) by a combination of experimental antithymocyte therapy, orbital radiotherapy with high-dose steroids.

**Methods:**

A 72-year-old man presented with severe vision loss in both eyes. The visual symptoms had appeared over a year before the GO diagnosis. He was initially misdiagnosed with neuroborreliosis and optic neuritis based on brain and orbital magnetic resonance imaging. There was no exophthalmos. The ophthalmological examination included visual acuity, visual field, tonometry in primary and upgaze eye position, optical coherence tomography (OCT), pattern electroretinogram (PERG), pattern, and flash visual evoked potentials (PVEP and FVEP). The patient received experimental therapy with ATG, followed by high-dose of intravenous steroids and orbital radiotherapy.

**Results:**

Delayed VEP peaks became shorter after treatment. After systemic and local therapy lowering of intraocular pressure was achieved. Abnormal PERG has been found three months before ganglion cells atrophy was detected in OCT. Visual acuity and visual field improvement occurred in both eyes after therapy, despite partial left optic nerve atrophy. The patient regained full decimal visual acuity (1.0 right from as poor as 0.3  to 1.0 in the right eye and from hand movements to 0.9 in the left. Severe visual field loss with advanced absolute scotomata has improved to slight relative scotomata. The duration of follow-up time after the treatment was 4 months.

**Conclusions:**

Intensive treatment of steroid-resistant Graves’ orbitopathy (GO) may prevent total optic nerve atrophy. Despite severely advanced optic neuropathy, this report emphasizes the necessity of therapy even with nearly complete visual function loss hence there is always a possibility to regain full visual acuity and visual field. Patients with tense orbital septum may not present with significant exophthalmos, thus delaying the correct diagnosis of orbitopathy. A supporting sign of GO was the difference in intraocular pressure in the primary and upgaze eye positions. Electrophysiological examinations are helpful in the diagnosis and monitoring of GO therapy. To our knowledge, this is the first report of this kind presenting visual function restoration and structural recovery in a patient with advanced optic neuropathy in GO.

## Introduction

Thyroid eye disease (TED), also known as Graves’ orbitopathy (GO) is an autoimmune mediated disease that can induce dysthyroid compressive optic neuropathy (DON). Traditional treatment of the moderate-to-severe active GO includes intravenous methylprednisolone (or in combination with mycophenolate sodium since 2021). Steroid-resistant orbitopathy is a major challenge and includes the second course of intravenous methylprednisolone, oral prednisone/prednisolone combined with either cyclosporine A or azathioprine; orbital radiotherapy combined with oral or intravenous glucocorticoids, teprotumumab; rituximab and tocilizumab. Sight-threatening GO is treated with high-doses of intravenous methylprednisolone and, if unresponsive, with urgent orbital decompression [1]. All these treatment options possess significant limitations.

Thymoglobulin (ATG) is a polyclonal rabbit antibody that causes T-cell depletion, used in the induction after kidney transplantation and treatment of acute graft rejection episodes. In addition, ATG in vitro induces apoptosis of naive plasma B cells and plasma cells [2], inhibits the secondary immune response by memory B cells via T-cell modulation, and induces regulatory T cells during immune reconstitution [3]; hence, it may suppress B cells and antibodies production. Experimental studies showed a positive impact of ATG in the management of active steroid-resistant GO [4, 5].

During the active phase of GO, orbital tissues are infiltrated by mononuclear cells, mainly lymphocytes, and plasmatic cells which produce antibodies, mast cells, and macrophages. Cumulation of aminoglycosides leads to oculomotor muscle enlargement and dysfunction. The proliferation of fat tissue increases the volume of orbital tissues causing exophthalmos, oculomotor muscles dysfunction, hyperemia and edema of orbital tissues, and compressive optic neuropathy.

The authors report a reversal of asymmetrical bilateral dysthyroid compressive optic neuropathy, more advanced in the left eye, managed with high-dose steroids, antithymocyte antibodies, and orbital radiotherapy.

## Case report

We present a diagnostic and therapeutic process in the difficult case of compressive optic neuropathy with severe vision loss due to Graves’ orbitopathy.

A 71-year-old man presented as an active smoker for more than 20 years with a history of type 2 diabetes mellitus (treated with metformin), hypertension, prostatectomy, and radiation therapy for prostate cancer, was treated with thyroxine for 15 years for hypothyroidism due to chronic autoimmune thyroiditis. First eye symptoms appeared in 2018 when the patient noticed double vision in an upgaze. In the course of their diagnosis in July 2018, Lyme disease was diagnosed, after which he received doxycycline with no subjective improvement. In January 2019 the patient was admitted to the department of infectious diseases, where neuroborreliosis was not confirmed. Magnetic resonance imaging (MRI) of the head revealed vascular changes and enlargement of inferior and superior recti muscles of the left eye. The consulting ophthalmologist administered a tapering dose of methylprednisolone from 32 mg with a reduction to half doses every 7 days. Additionally, ceftriaxone was ordered for 30 days. Due to persistent ophthalmic symptoms, in March 2019 the patient was again hospitalized for borreliosis as a preliminary diagnosis. However, observation toward the involvement of the central nervous system was negative. No improvement in terms of diplopia was achieved. According to an ophthalmologist, methylprednisolone from 16 mg with gradual dose reduction was again instituted. We were not provided with ophthalmic records regarding best corrected visual acuity (BCVA) and intraocular pressure (IOP). Altogether patient received 1 g of methylprednisolone during both hospitalizations.

Subsequently, in July 2019 the patient was admitted to the ophthalmology ward on account of severe pain in the left eye (LE) and color vision disturbances, as colors seemed washed out. Diagnosis of left eye posterior optic neuritis was made. Repeatedly, intravenous methylprednisolone followed by an oral route was administered. No detailed medical records or documentation on the dose were available.

**Fig. 1:**
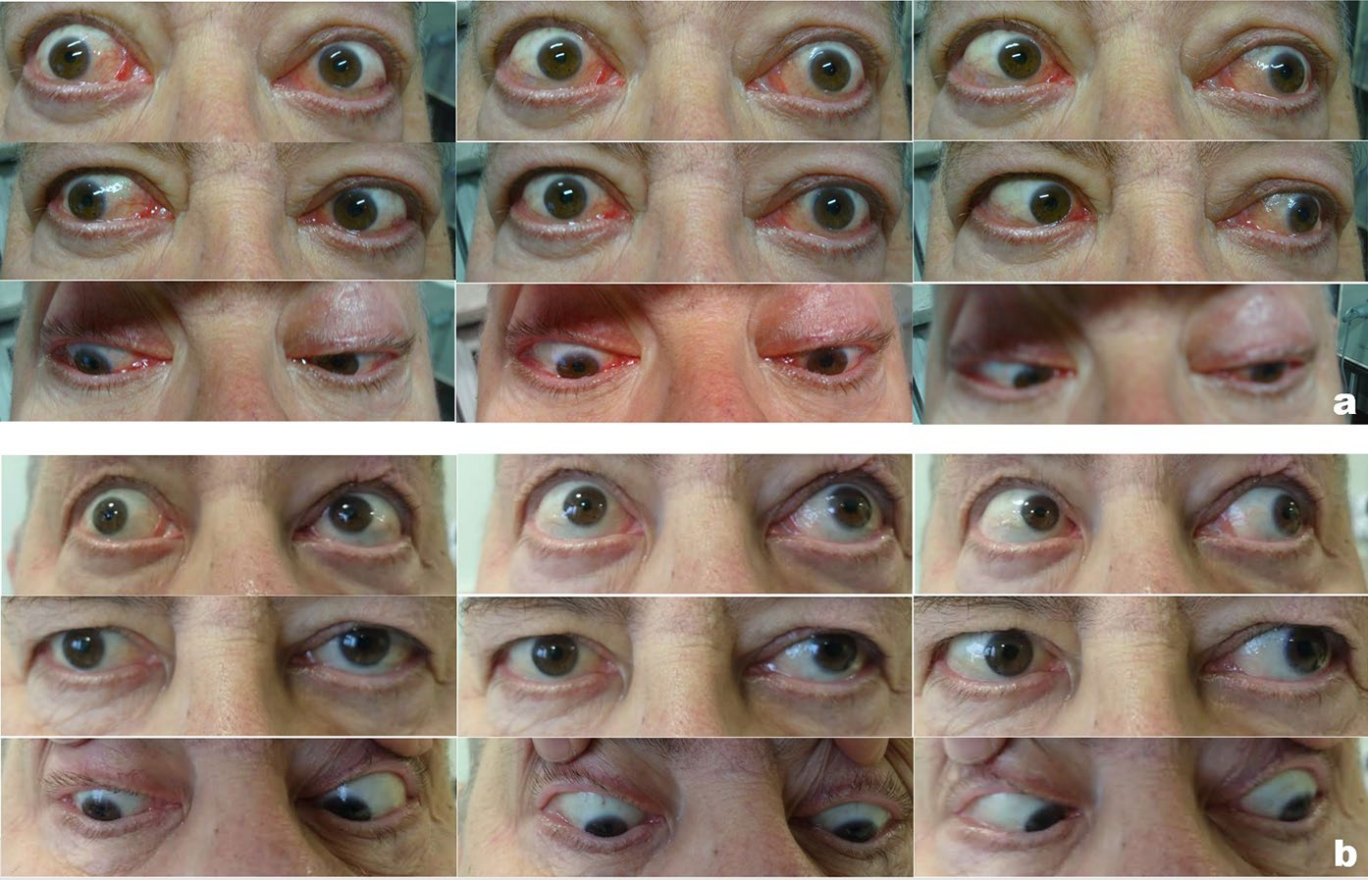
Ocular motility **a** before treatment, in the active phase, **b** inactive phase

Due to progressive visual acuity loss, the patient was referred to our department of ophthalmology (10.09.2019). This was our first contact with the patient and from this date, we have detailed ophthalmic records. Decimal best corrected visual acuity (BCVA) on Snellen chart was 1.0 in the right eye (RE) and 0.3 in the LE. Severe color vision loss was revealed (Ishihara plates, Japan). Exophthalmos with Hertel’s exophthalmometer at the level of 25 mm for each eye was detected. However, its presence was partially masked by eyelid edema and floppiness of periocular skin hindering the diagnosis of Graves’ orbitopathy (Fig. 1).

**Fig. 2:**
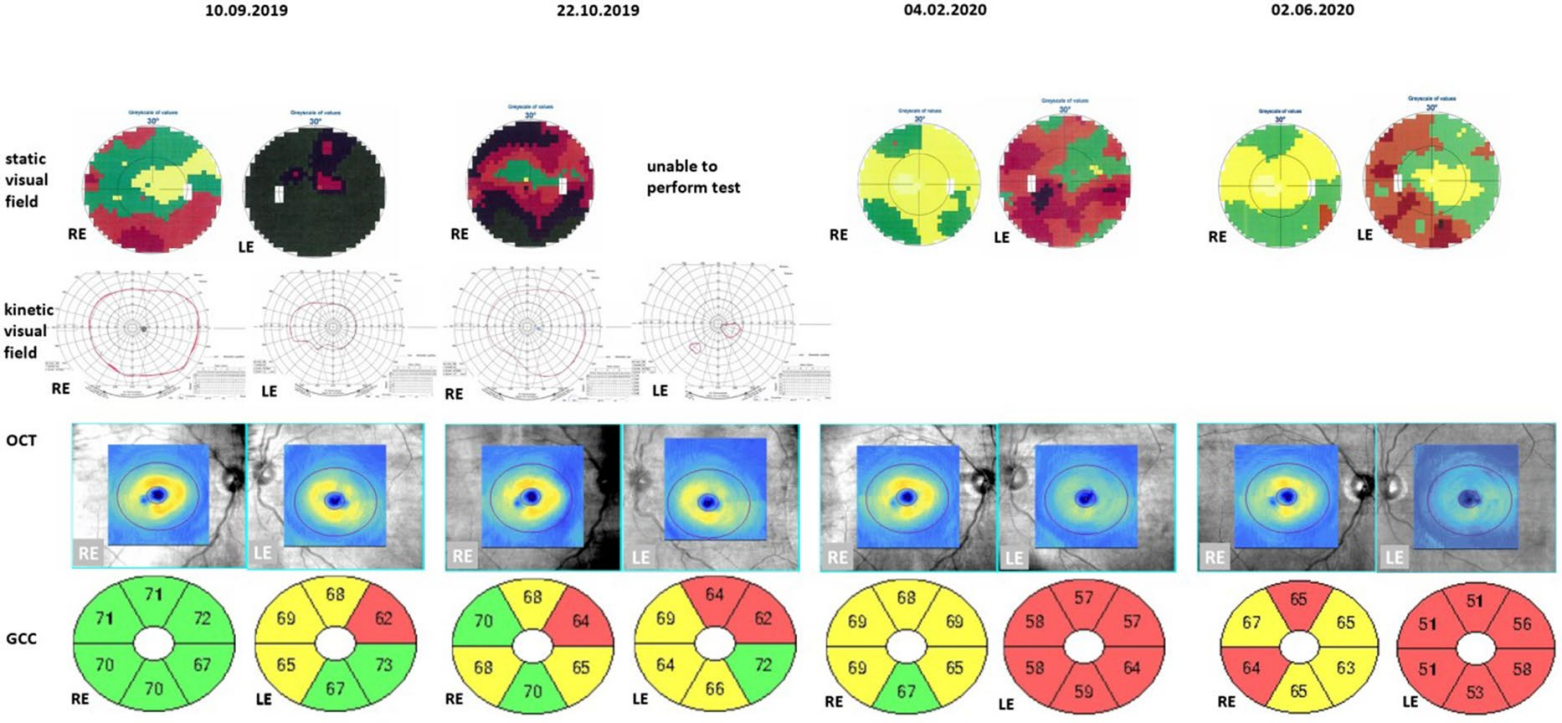
Visual field and GCC loss in OCT in the course of the disease

**Fig. 3:**
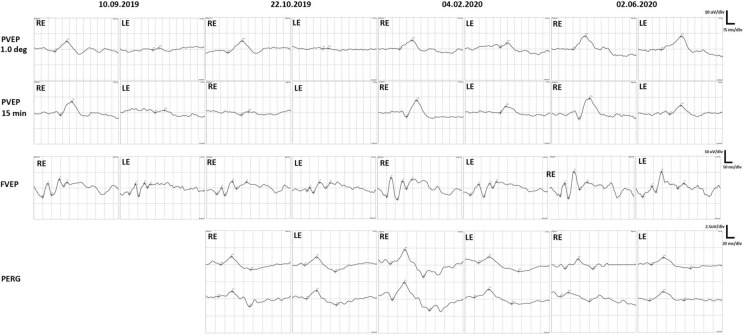
Electrophysiology tests, PVEP, FVEP and PERG, in the observational period

Differences in intraocular pressure (Kowa applanation tonometer HA-2, Japan) between the upgaze (RE 23 mmHg, LE 27 mmHg) and primary gaze positions (RE 12 mmHg, LE 15 mmHg) were noticed. In the RE IOP increased by 191% and in the LE rose by 180% from the primary to upgaze position (Table 1). The patient received topical timolol and dorzolamide twice a day to both eyes to lower IOP. Static visual field (Octopus 600, Haag-Streit, Switzerland) revealed advanced relative scotomata in the RE and absolute scotomata in the LE. Goldman kinetic perimetry (Campus, Haag-Streit, Switzerland) showed constriction of the visual field on the LE. Optical coherent tomography (Zeiss, Cirrus 6000, Germany) presented reduced ganglion cell complex (GCC) in the LE (Fig. 2). Diabetic retinopathy and diabetic macular edema were not present in dilated fundus examination. Pattern and flash visual evoked potentials (PVEP/FVEP) (Reti-Port electrophysiological device, Roland Consult, Germany) were recorded following the standards of the International Society of Clinical Electrophysiology of Vision (ISCEV) [6, 7], (Tables 2, 3, Fig. 3). In the PVEP significantly prolonged peak latency and reduced amplitude of P100 wave were found. The patient was assessed with a clinical activity scale (CAS) and NOSPECS scale (Table 1). Compressive optic neuropathy was recognized. On this basis, the diagnosis of GO was made. The patient was immediately referred to the endocrinology department, where the diagnosis was confirmed (positive TRAb).

**Table 1:** BCVA, IOP, exopthalmometry, CAS, NOSPECS and ocular motility before treatment and during follow-up visits

		Before treatment 10.09.2019	During treatment (ATG) 22.10.2019	End of the treatment 04.02.2020	Follow-up 02.06.2020
Decimal BCVA RE		1.0	0.3	1.0	1.0
Decimal BCVA LE		0.3 (neuropathy and keratopathy)	Hand movements (lazy pupil reaction to the light; optic nerve edema)	0.9	0.8 (cataract)
*IOP [mmHg]*					
RE–primary eye gaze position		12	17	14	13
– upgaze		23	27	20	21
*IOP [mmHg]*					
LE–primary eye gaze position		15	19	14	15
– upgaze		27	31	21	21
Antiglaucoma medications–RE		dorzolamide 2x timolol 2x	dorzolamide 2x timolol 2x brimonidine 2x	dorzolamide 2x timolol 2x brimonidine 2x	dorzolamide 2x timolol 2x brimonidine 2x
Antiglaucoma medications–LE		dorzolamide 2x timolol 2x latanoprost 1x	dorzolamide 2x timolol 2x brimonidine 2x	dorzolamide 2x timolol 2x brimonidine 2x	dorzolamide 2x timolol 2x brimonidine 2x
Systemic antiglaucoma medications			acetazolamide 1 × 125 mg		
Exophthalmometry RE [mm]		25	25	22	20.5
Exophthalmometry LE [mm]		24	25	22	21.5
NOSPECS		2-b, 3-b, 4-b, 5-a, 6-b	2-b, 3-b, 4-c, 5-a, 6-c	2-a, 3-a, 4-c, 5-a, 6-b	2–0, 3-a, 4-a, 5-a, 6-a
CAS		6	9	2	2
Ocular motility [degrees]	RE	15°up	0° up	10° up	18° up
(references		48° temporal	38° temporal	53° temporal	37° temporal
		38° nasal	30° nasal	45° nasal	45° nasal
up–50°		55°down	50° down	55° down	65° down
down–70°	LE	10° up	0° up	10° up	19° up
nasal–50°		35° nasal	12° nasal	40° nasal	40° nasal
		45° temporal	28° temporal	55° temporal	45° temporal
temporal–90°)		55° down	25° down	55° down	65° down

**Table 2:** Pattern VEP before treatment and during follow-up visits

Pattern VEP	Large checks (1°)				Small checks (15’)			
	RE		LE		RE		LE	
Date	PkL [ms]	A [µV]	PkL [ms]	A [µV]	PkL [ms]	A [µV]	PkL [ms]	A [µV]
10.09.2019	116.8	6.6	135	1.5	134.4	8.7	152	1.1
22.10.2019	129.7	7.5	123.9	0.8	149.1	2.1	Non-recordable	
04.02.2020	119.8	5.8	149.7	2.8	135	10.5	143.8	4.3
02.06.2020	119.2	8.4	152.6	8.5	135	13.2	152.6	5.5
Reference values Mean ± SD	103 ± 10	8.1 ± 1.1	103 ± 10	8.1 ± 1.1	108 ± 13	7.8 ± 1.6	108 ± 13	7.8 ± 1.6

*A*—Amplitude measured from the preceding trough; *PkL*—P100 peak Latency; *SD*—Standard deviation

**Table 3:** Flash VEP before treatment and during follow-up visits

Flash VEP	RE				LE			
	P1		P2		P1		P2	
Date	PkL [ms]	A [µV]	PkL [ms]	A [µV]	PkL [ms]	A [µV]	PkL [ms]	A [µV]
10.09.2019	79.8	8.67	145.6	10.1	88.3	6.5	139.0	8.4
22.10.2019	86.4	4.2	148.4	8.9	102.4	2.4	157.8	6.7
04.02.2020	78.0	12.8	142.8	11.8	88.3	7.1	151.2	8.7
02.06.2020	87.4	8.2	139.0	16.9	92.1	6.9	145.6	13

*A*—Amplitude measured from the preceding trough; *PkL*—Latency

Just one and half months later (22.10.2019) the patient developed absolute scotomata in the RE eye. It was impossible to obtain a visual field of the LE, due to the severe progression of compressive optic neuropathy. Prolonged latency and lower amplitudes of P100 and P2 in both eyes (more advanced in LE) were signs of compressive and ischemic optic neuropathy consecutively.

PERG (DTL electrodes, Reti-Port electrophysiological device, Roland Consult, Germany) recorded following the ISCEV standards [6, 7] (Fig. 3, Table 4) significantly revealed a progressive loss of P50 and N95 amplitudes in both eyes, which was associated with abnormalities in the structural loss of GCC in OCT results.

**Table 4:** Pattern ERG before treatment and during follow-up visits

Pattern ERG	RE				LE				RE	LE
	P50		N95		P50		N95		N95/P50 ratio	N95/P50 ratio
Date	PT[ms]	A[µV]	PT[ms]	A[µV]	PT[ms]	A[µV]	PT[ms]	A[µV]		
22.10.2019	54.6	1.1	95.8	1.9	51.4	1.2	90.9	2.1	1.72	1.75
04.02.2020	55.7	2.1	95.0	4	51.4	1	99.7	2.1	1.90	2.10
02.06.2020	56.0	1.4	88.8	1.4	53.9	1.3	99.0	1.6	1.0	1.23
Reference values Mean ± SD	50 ± 10	4 ± 2	95 ± 10	7 ± 2	50 ± 10	4 ± 2	95 ± 10	7 ± 2	1.75–2.0	1.75–2.0

*A*—Amplitude; *PT*—Peak time; *SD*—Standard deviation

Experimental intravenous ATG therapy was administered twice 100 mg per dosage, as part of a clinical trial conducted with the approval of the Bioethics Committee of the Silesian Medical University.

Despite the experimental treatment, during one and half months (December 2019) further vision loss led to intravenous methylprednisolone administration of up to 8 g of total intravenous dose, followed by oral route. Additional orbital radiotherapy, delivered in 10 fractions with a cumulative dosage of 20 Gy contributed to visual acuity and visual field improvement. At the end of the treatment (04.02.2020) patient’s visual acuity improved to 1.0 in the RE and to 0.9 in the LE.

On the follow-up visit (02.06.2020) ocular motility is still abnormal after treatment but double vision occurred only in extremes of gaze.

**Fig. 4:**
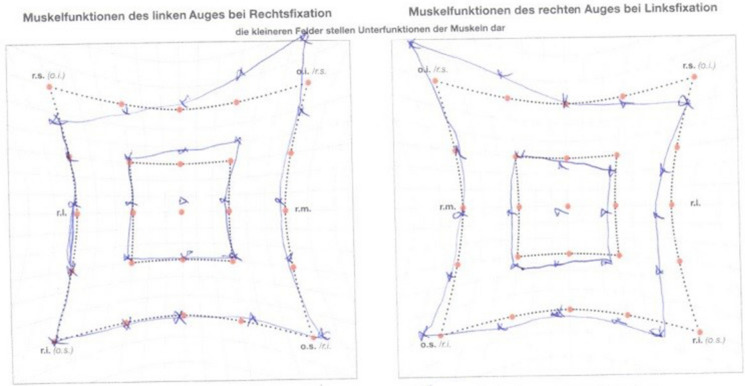
Hess charts indicating residual ocular muscles disfunction

The patient’s ocular motility was assessed with a Hess chart (Fig. 4). The test revealed limitation of superior rectus muscle function in both eyes, probably secondary to fibrosis of the inferior rectus muscle with overaction of the inferior oblique muscle. Additionally, restriction of abduction was found in the right eye. A comparison of the patient’s eyes and ocular motility in the active phase of TED and after successful treatment is shown in Fig. 1. GCC loss finally occurred in both eyes despite successive treatment. Loss and recovery of PVEP are presented in Fig. 3.

## Discussion

In ophthalmic practice, initially, most cases of GO require no special treatment. However, in patients with a severe and steroid-resistant course of the disease, additional treatment is mandatory.

Despite many reports in the literature, the problem of insufficient diagnosis of this disease is still very common in clinical practice and should be highlighted.

In the present patient the first signs and symptoms of TED had started a year before the correct diagnosis was made. Delay in the diagnosis was caused by the slow progression of the disease and an absence of severe proptosis, which was masked by eyelid edema and floppiness of periorbital skin. In some individuals, severe orbitopathy is present despite a slight or lack of exophthalmos due to the tense orbital septum and lack of its relaxation, which prevents self-decompression (eye globe proptosis) [8]. The disease may evolve into a severe form with nearly complete vision loss in the left eye, highlighting the need for close observation of patients with TED, despite intensive steroid therapy [9].

Accurate assessment of the clinical features of TED is crucial for early diagnosis of the high-risk disease, planning medical and surgical strategies, and evaluating response to therapy [10]. Lack of appropriate therapy for DON may lead to further worsening of vision including blindness and other ophthalmic complications [11].

GO is most common in Graves’ disease; however, severe cases of orbitopathy have been described in patients with Hashimoto’s thyroiditis [12]. The presented patient had initially Hashimoto’s disease which transformed into Graves’ disease.

Diagnostic delay of Graves’ orbitopathy results in significant morbidity and increases patient dissatisfaction. However, it can be challenging even for ophthalmologists to recognize orbitopathy. In the presented case misdiagnoses with borreliosis, and optic neuritis contributed to the late diagnosis.

In thyroid eye disease prolonged positive peak latencies (P100 in PVEP and P2 in FVEP) enabled control of the course of compressive optic neuropathy [13]. In our patient prolonged latencies in pattern and flash VEPs were noted in the active phase, has reduced after treatment.

Inflammation-induced enlargement of ocular muscles compresses the eye globe and can cause intraocular pressure to rise in the primary position. Moreover, restrictive fibrosis of the inferior rectus muscle can additionally increase the intraocular pressure in the upgaze position [14].

In the presented case, in the primary eye position the pressure was 18 mmHg but in upgaze increased to 30 mmHg. Systemic treatment reduced muscle inflammation and normalized intraocular pressure by decreasing production and improving aqueous outflow.

Optic neuropathy in GO is caused by compression of the optic nerve by orbital tissues as well as compression of the eye globe by ocular muscles and by complications of steroid therapy, which both led to secondary glaucoma. Glaucoma treatment is a very important part of therapy.

Ambrosio et al. revealed the clinical usefulness of VEPs in the differential diagnosis of patients with ophthalmopathy and ocular hypertension or suspect glaucoma with dysthyroid optic neuropathy patients [15].

Significant ganglion cell loss was found in OCT in February 2020, while abnormal PERG was detected in October 2019. PERG was abnormal in both eyes from the beginning of observation. Amplitudes of P50 and N95 were lower than normal. The N95/P50 ratio was abnormal in the RE and very low in the LE. It improved during the treatment but finally remained abnormal in both eyes. This means that optic atrophy was more advanced than indicated by the visual field and OCT examination. The PERG arises largely in the ganglion cells, P50 is driven by the photoreceptors and corresponding retinal cells in the macular area, while N95 originates from macular ganglion cells which are located more proximally. Lower than normal N95 amplitude and N95/P50 ratio were found in the early stage of optic atrophy. In advanced optic atrophy, both P50 and N95 present with low amplitudes.

Functional changes precede morphological changes, thus tests that assess the function of GCC should be done before its loss. Abnormal Pattern ERG test preceded by three months of the loss of GCC found in OCT. Treatment decisions should be also made based on functional tests.

## Conclusions

Intensive treatment of steroid-resistant GO may prevent total optic nerve atrophy. Despite severe, advanced optic neuropathy, this report emphasizes the necessity of therapy even with nearly complete visual function loss. Hence there is always a possibility to regain full visual acuity and visual field. Patients with tense orbital septum may not present with significant exophthalmos, thus delaying the correct diagnosis of orbitopathy. A supporting sign of GO was the difference in intraocular pressure in the primary and in upgaze eye positions. Electrophysiological examinations in GO are helpful tools in the diagnostic and therapeutic process.

To our knowledge, this is the first report of this kind presenting such a remarkable visual function recovery in a patient with advanced optic neuropathy in Graves’ orbitopathy.

However, whether only steroids or a combination of ATG, orbital radiotherapy with steroids caused improvement cannot be determined based on a single case report.

## Data Availability

All data and materials are available in the manuscript.

## Declarations

### Statement on the welfare of animals

This article does not contain any studies with animals performed by any of the authors.
